# The risk of miscarriage following surgical treatment of heterotopic extrauterine pregnancies

**DOI:** 10.1093/hropen/hoab046

**Published:** 2022-01-03

**Authors:** S A Solangon, M Otify, J Gaughran, T Holland, J Ross, D Jurkovic

**Affiliations:** Gynaecology Diagnostics and Outpatient Treatment Unit, University College London Hospital, London, UK; Early Pregnancy and Gynaecology Unit, King’s College Hospital, London, UK; Liverpool Women’s Hospital, Liverpool, UK; Early Pregnancy and Gynaecology Unit, Guy’s and St Thomas’ Hospital, London, UK; Early Pregnancy and Gynaecology Unit, Guy’s and St Thomas’ Hospital, London, UK; Early Pregnancy and Gynaecology Unit, King’s College Hospital, London, UK; Gynaecology Diagnostics and Outpatient Treatment Unit, University College London Hospital, London, UK; Institute for Women’s Health, University College London, London, UK

**Keywords:** pregnancy ectopic, pregnancy heterotopic, abortion spontaneous, laparoscopic surgery, salpingectomy

## Abstract

**STUDY QUESTION:**

What is the risk of loss of a live normally sited (eutopic) pregnancy following surgical treatment of the concomitant extrauterine ectopic pregnancy?

**SUMMARY ANSWER:**

In women diagnosed with heterotopic pregnancies, minimally invasive surgery to treat the extrauterine ectopic pregnancy does not increase the risk of miscarriage of the concomitant live eutopic pregnancy.

**WHAT IS KNOWN ALREADY:**

Previous studies have indicated that surgical treatment of the concomitant ectopic pregnancy in women with live eutopic pregnancies could be associated with an increased risk of miscarriage. The findings of our study did not confirm that.

**STUDY DESIGN, SIZE, DURATION:**

A retrospective observational case–control study of 52 women diagnosed with live eutopic and concomitant extrauterine pregnancies matched to 156 women with live normally sited singleton pregnancies. The study was carried out in three London early pregnancy units (EPUs) covering a 20-year period between April 2000 and November 2019.

**PARTICIPANTS/MATERIALS, SETTING, METHODS:**

All women attended EPUs because of suspected early pregnancy complications. The diagnosis of heterotopic pregnancy was made on ultrasound scan and women were subsequently offered surgical or expectant management.

There were three controls per each case who were randomly selected from our clinical database and were matched for maternal age, mode of conception and gestational age at presentation.

**MAIN RESULTS AND THE ROLE OF CHANCE:**

In the study group 49/52 (94%) women had surgery and 3/52 (6%) were managed expectantly. There were 9/52 (17%, 95% CI 8.2–30.3) miscarriages <12 weeks’ gestation and 9/49 (18%, 95% CI 8.7–32) miscarriages in those treated surgically. In the control group, there were 28/156 (18%, 95% CI 12.2–24.8) miscarriages <12 weeks’ gestation, which was not significantly different from heterotopic pregnancies who were treated surgically [odds ratio (OR) 1.03 95% CI 0.44–2.36]. There was a further second trimester miscarriage in the study group and one in the control group. The live birth rate in the study group was 41/51 (80%, 95% CI 66.9–90.2) and 38/48 (79%, 95% CI 65–89.5) for those who were treated surgically. These results were similar to 127/156 (81%, 95% CI 74.4–87.2) live births in the control group (OR 0.87, 95% CI 0.39–1.94).

**LIMITATIONS, REASONS FOR CAUTION:**

This study is retrospective, and the number of patients is relatively small, which reflects the rarity of heterotopic pregnancies. Heterotopic pregnancies without a known outcome were excluded from analysis.

**WIDER IMPLICATIONS OF THE FINDINGS:**

This study demonstrates that in women diagnosed with heterotopic pregnancies, minimally invasive surgery to treat the extrauterine pregnancy does not increase the risk of miscarriage of the concomitant live eutopic pregnancy. This finding will be helpful to women and their clinicians when discussing the options for treating heterotopic pregnancies.

**STUDY FUNDING/COMPETING INTEREST(S):**

This work did not receive any funding. None of the authors has any conflict of interest to declare.

**TRIAL REGISTRATION NUMBER:**

Research Registry: researchregistry6430.

WHAT DOES THIS MEAN FOR PATIENTS?

This study is about heterotopic pregnancies, which is when a woman has a twin pregnancy, one of which is normally sited inside the womb and the other is an ectopic pregnancy growing outside the womb. Ectopic pregnancies can endanger the mother’s health and most of them have to be surgically removed. Surgery to treat ectopic pregnancies is generally safe for mothers but it is not known whether an operation may harm normal healthy pregnancies inside the womb causing them to miscarry.

Some previous studies have suggested that there is a higher risk of miscarriage following surgery for the ectopic twin. However, we are not completely certain about that as some studies have also included normally sited pregnancies, which had already miscarried before surgery was carried out. In view of that, we were not able to tell women diagnosed with heterotopic pregnancies how likely it is that surgery to treat ectopic could cause them to lose their live normally sited pregnancy.

Our study was conducted in three large hospitals, and we only included women with heterotopic pregnancies who had a live normally sited pregnancy. We collected information about the number of pregnancies that miscarried in the first 3 months and the number of pregnancies which resulted in the delivery of a healthy baby. We found that miscarriage rates were not higher in women who had surgery for heterotopic pregnancy compared to those who had a live singleton normally sited pregnancy and did not have any surgical intervention.

Our results will be of great help to women diagnosed with heterotopic pregnancies who could be reassured that surgery to remove the ectopic twin is probably not going to harm the other pregnancy developing inside the womb.

## Introduction

A heterotopic pregnancy (HP) is defined as the simultaneous occurrence of a normally sited (eutopic) pregnancy and an ectopic pregnancy (ESHRE working group on Ectopic Pregnancy, 2020). The presence of a normally sited pregnancy is often perceived as a reassuring sign by clinicians when assessing women presenting with clinical signs suspicious of ectopic pregnancy. This can lead to a delay in diagnosing the concomitant ectopic pregnancy with an increased risk of adverse maternal outcomes. The concomitant ectopic pregnancy can occur in any location outside the uterine cavity. The most common sites are the Fallopian tubes, uterine Caesarean section scar defect and ovaries.

Historically, HP had been reported to occur in 0.003% (1:30 000) spontaneous pregnancies and in 1% of pregnancies conceived via ART (Svare *et al.*, 1993; Tal *et al.*, 1996; Li*et al.*, 2013). HP used to be more common in pregnancies conceived through ART due to the previous more prevalent practice of transferring more than one embryo. Women undergoing ART are also more likely to have tubal pathology, thereby increasing their risk of an ectopic pregnancy (Parashi *et al.*, 2014; Li *et al.*, 2015). Factors that may compromise tubal patency include a previous ectopic pregnancy (Jeon *et al.*, 2016), tubal surgery (Lv *et al.*, 2020), pelvic surgery (Malak *et al.*, 2011), pelvic inflammatory disease (Xiao *et al.*, 2018) and hydrosalpinges (Liu *et al.*, 2015). Failed tubal sterilization and intrauterine devices can also increase the risk of an ectopic pregnancy (Li *et al.*, 2015). More recent reports from the UK showed a prevalence of 0.05% (1:2000) in women attending early pregnancy units (EPUs) (Dooley *et al.*, 2021) and 0.04% (1:2500) in ART pregnancies (Santos-Ribeiro *et al.*, 2016), probably reflecting the move towards single embryo transfers.

The ectopic pregnancy in an HP can be treated surgically or by ultrasound-guided aspiration, with the aim of preserving the concomitant live eutopic pregnancy. The surgical method is dependent on the location of the ectopic pregnancy and in cases of tubal ectopics usually involves laparoscopic salpingectomy (Soriano *et al.*, 2010). If the eutopic pregnancy has already miscarried, conservative surgery can be considered such as expectant or medical treatment with methotrexate (Yu *et al.*, 2014). In cases of uterine ectopics (caesarean section scar, cervical or intramural), a complete surgical removal of ectopic pregnancy is not often possible without damaging the eutopic pregnancy and ultrasound-guided embryocide is usually the only available treatment option.

Previous studies of HP have reported widely varying rates of miscarriage and live birth rates of concomitant eutopic pregnancies (Barrenetxea *et al.*, 2007; Clayton*et al.*, 2007; Talbot *et al.*, 2011; Li *et al.*, 2016; Lyu *et al.*, 2017). This could be explained by the heterogeneity of definitions of pregnancy viability and reported outcomes used by different authors. Many studies have not discriminated between the outcomes of eutopic pregnancies that were live or not live at the time of diagnosis, with only a few of them carried out as case-controlled observations. There is no robust data on whether treatment of HP affects the outcome of a live eutopic pregnancy, making it difficult to advise women about the risks associated with surgical treatment of ectopic pregnancy. The aim of our study was to examine the risk of miscarriage of live eutopic pregnancies following minimally invasive surgical treatment of the concomitant ectopic pregnancy.

## Materials and methods

This was a retrospective observational case–control study of HPs carried out in three London specialist EPUs between April 2000 and November 2019. Patients had either presented to an EPU of their own accord or were referred from general practitioners, private fertility clinics or other hospitals. Clinical information, ultrasound images, operation notes and histological results were retrieved from dedicated hospital clinical databases. Data were anonymized and securely stored according to General Data Protection Regulations (GDPR).

HPs were diagnosed on ultrasound based on direct visualization of an ectopic pregnancy concomitant with an eutopic pregnancy. Ultrasound scans were performed by clinical fellows, sonographers and midwives who were either under direct supervision or supported by specialist gynaecologists with high expertise in diagnosing and managing early pregnancy complications.

A live pregnancy was defined by the presence of an embryo/foetus with visible cardiac activity on ultrasound scan. Pregnancies that were less than 6 weeks’ gestation were considered potentially live if the morphological appearances on ultrasound scan were consistent with the gestational age.

Miscarriage was classified as (i) early embryonic demise in women with an intact gestation sac ≥25 mm in size without an embryo or with an embryo measuring ≥7 mm with no evidence of cardiac activity or if the pregnancy had not progressed as expected over a period of follow-up, with a minimum interval between the visits of 7 days, (ii) incomplete miscarriage defined by the presence of solid, hyperechoic, poorly defined and vascular echogenic tissue within the uterine cavity and (iii) complete miscarriage in the presence of an empty uterine cavity in women with previous conclusive evidence of a normally sited pregnancy.

Ectopic pregnancies were defined as pregnancies located partially or completely outside the uterine cavity and included pregnancies in the Fallopian tubes, ovaries, abdominal cavity, caesarean section scar and the cervix. Interstitial pregnancies were classified as tubal pregnancies, as per ESHRE definition. Pregnancies growing in a non-communicating rudimentary horn of a unicornuate uterus were also classified as ectopics (ESHRE working group on Ectopic Pregnancy, 2020).

The ectopic pregnancies were further classified according to their morphological characteristics into five groups: gestational sac with an embryo and cardiac activity (Type I), gestational sac with an embryo and no cardiac activity (Type II), gestational sac with a yolk sac (YS) and no embryo (Type III), empty gestational sac (Type IV) or a solid, homogenous mass (Type V).

We identified all women who had an HP and included those with live or potentially live eutopic pregnancies at the time of diagnosis. We excluded women who did not have follow-up after 12 weeks’ gestation and those who opted for termination of the eutopic pregnancy. HPs which were located within the cervix or caesarean section scar were also excluded from the final analysis due to differences in treatment between uterine and extrauterine ectopic pregnancies. In women who underwent surgery, the diagnosis of ectopic pregnancy was confirmed at laparoscopy and on histological examination of surgical specimens.

We also identified three normal pregnancies (controls) per HP case, which were matched for maternal age, mode of conception and gestational age at presentation. The latter was calculated by the last menstrual period or date of conception from oocyte retrieval and fertilization. Controls were randomly selected from a database of all early pregnancy attendances to the EPU and Reproductive Medicine Unit (RMU) at University College London Hospital (UCLH) between 2005 and 2019. Cases selected all had initial scans showing a live or potentially live eutopic pregnancy. We used an on-line computer random number generator (random.org) to select the control cases, in order to eliminate selection bias and to balance the two groups.

The primary outcome was the pregnancy outcome at 12 weeks’ gestation in the study and control group. Secondary outcome included the final pregnancy outcome. Sub-analysis was undertaken for pregnancy outcome according to treatment of HP. The number and percentage in each category were calculated for categorical variables. The baseline variables for normality of distribution were tested using the Shapiro–Wilk test. The mean and SD were calculated for normally distributed continuous variables. The median and interquartile ranges were calculated for continuous variables that were not normally distributed. Categorical variables with no ordering to the categories were analysed using the Chi-square test, except for when there were small numbers in some groups, where Fisher’s exact test was preferred. Mann–Whitney test was used to compare groups when there was a natural order to the categories. Continuous variables were compared between groups using the unpaired *t*-test. Odds ratio (OR) and CI were determined and a *P*-value of <0.05 was considered statistically significant. The data were analysed using logistic regression that firstly examined the association between factors and outcome in a series of univariable analyses. Subsequently, the joint association between the factors and outcome was examined in a multivariable analysis. Only those with a *P*-value of <0.2 from the univariable analyses were included in the second stage to limit the number of factors. A backwards selection procedure was used to retain only those factors associated with the outcome in the final model. This involves omitting non-significant variables, one at a time until all variables are significant (or close to significance). Statistical analyses were performed using Stata for Windows.

We estimated that we would need a minimum of 50 women with HP assuming the background miscarriage rate of 12% (Magnus *et al.*, 2019) and the HP miscarriage rate of 31% in HP (Barrenetxea *et al.*, 2007), to show a statistically significant difference between the two groups with a confidence level of 95% and 80% power. We sought advice from the National Health Service Research Ethics Committee and the Joint Research Office at UCLH regarding ethical approval and were advised that formal ethics approval was not needed for this study as the data had already been collected as part of routine care, and the data were anonymized and analysed within the care team. This study was registered with the Research Registry with the unique identifying number: researchregistry6430.

## Results

During the study period, 92 women were diagnosed with heterotopic pregnancies in three participating units. There were 84 women with concomitant eutopic and extrauterine pregnancies and eight with concomitant uterine ectopic pregnancies. In the subgroup of women with extrauterine heterotopic pregnancies, a live eutopic pregnancy was present on the initial scan in 67/84 (80%) cases, whilst the remaining women were diagnosed with miscarriages. We excluded women who did not complete follow-up at the three participating EPUs and those who had elective termination of pregnancy. This left 52 women with live eutopic and concomitant extrauterine ectopic pregnancies included in the final analysis. We also identified 156 matched control cases.

Maternal demographic characteristics are shown in Table I. The study and the control group were matched for maternal age, mode of conception and gestational age at presentation. Gravidity and parity were also similar.

**Table I:** Demographic characteristics of study patients and controls.

		HP (N = 52)	Controls (N = 156)
Median age* (range)		33.5 (21–47)	34 (21–47)
Median gestational age^#^ (range)		6^+6^ (4^+6^–14^+1^)	6^+6^ (4^+6^–14^+1^)
ART (N%)		30 (57.7)	90 (57.7)
Parity (N%)	0	28 (53.8)	103 (66)
	1	17 (32.7)	39 (25)
	2+	7 (13.4)	14 (9)
Gravidity (N%)	1	14 (26.9)	61 (39.1)
	2	14 (26.9)	41 (26.3)
	3	9 (13.5)	24 (15.4)
	4+	15 (28.8)	30 (19.2)

HP, heterotopic pregnancy.

* Years.

^#^ Weeks and days.

In 49/52 (94%) of HP cases, the ectopic pregnancy was located within the Fallopian tube and in 3/52 (6%) within the ovary. The majority of ectopic pregnancies [49/52 (94%)] were treated by surgery (Table II). There was ultrasound evidence of haemoperitoneum in 17/49 (35%) women who required immediate surgery, 30/49 (61%) had no evidence of intra-abdominal bleeding, while in 2/49 (4%), the information on haemoperitoneum was missing.

**Table II:** Morphological types of extrauterine ectopic pregnancy and the type of treatment (N = 52).

	Surgical treatment			Expectant management	Total
Morphological type of extrauterine pregnancy^*^	Salpingectomy	Salpingotomy	Excision of ovarian ectopic		
I	14	2		0	16
II	2	1		0	3
III	4			1	5
IV	8		1	0	9
V	11		2	2	15
Unknown	4			0	4
Total	43	3	3	3	52

* Type I, gestational sac containing embryo with visible cardiac activity; Type II, gestational sac containing embryo with no visible cardiac activity; Type III, gestational sac containing only yolk sac with no visible embryo; Type IV, empty gestational sac with no visible additional structures; and Type V, solid inhomogeneous swelling.

Three tubal ectopic pregnancies were managed expectantly. None of them had evidence of haemoperitoneum or significant abdominal pain and they were all clinically stable. Two of these pregnancies were solid Type V ectopics. The third woman had a gestation sac and yolk sac, which developed an embryo with cardiac activity 1 week later. The embryonic heart rate eventually became bradycardic then absent over the monitoring period.

### Outcome at 12 weeks’ gestation

There were a total of 9/52 (17% 95% CI 8.2–30.3) miscarriages by 12 weeks’ gestation in the study group. In the subgroup of patients with HPs treated surgically, the miscarriage rate was 9/49 (18%, 95% CI 8.7–32) compared to 28/156 (18%, 95% CI 12.2–24.8) in the control group (OR 1.03 95% CI 0.44–2.36). In the subgroup of patients who had haemoperitoneum requiring immediate surgery, the miscarriage rate was 3/17 (18%, 95% CI 3.8–43) compared to 6/30 (20%, 95% CI 7.7–38.5) in those who did not have haemoperitoneum (OR 0.85, 95% CI 0.19–4.0).

### Final pregnancy outcome

Among pregnancies in the study group that progressed beyond 12 weeks’ gestation, the outcome was known in 42/43 cases. One woman did not attend for her follow-up visits after 22 weeks’ gestation, and the outcome of her pregnancy is unknown. One late loss occurred at 15 weeks’ gestation. The patient had a concomitant interstitial pregnancy and had a miscarriage a few days after the procedure. One late loss in the control group occurred at 18 weeks’ gestation after a spontaneous rupture of membranes due to chorioamnionitis.

The live birth rate in the study group was 41/51 (80%, 95% CI 66.9–90.2). In the subgroup of women who were treated surgically, 38/48 (79%, 95% CI 65–89.5) had live births compared to 127/156 (81%, 95% CI 74.4–87.2) in the control group (OR 0.87, 95% CI 0.39 to 1.94). In the subgroup of patients who had haemoperitoneum requiring immediate surgery, the live birth rate was 14/17 (82%, 95% CI 56.5–96.2) compared to 22/29 (76%, 95% CI 56.4–89.7) in those who did not have haemoperitoneum (OR 1.48, 95% CI 0.33–6.72).

There were no losses of normally sited pregnancies among women with HPs who were managed expectantly, but the numbers were too small to carry out meaningful statistical comparisons.

None of the outcomes of interest varied significantly between the study and the control group, even when adjusting for parity, gravidity and history of ectopic pregnancy.

### Logistic regression

Additional analyses examined the association between demographic and pre-pregnancy measures and outcome at 12 weeks’ gestation. Univariable associations in the control group are outlined in Table III. Parity was strongly associated with gravidity, and it was chosen not to adjust for it in the analyses. The results suggest that maternal age was significantly associated with outcome at 12 weeks’ gestation. Older women were at higher risk of miscarriage with every 5-year increase in age associated with a 2-fold increase in the odds of miscarriage. There was evidence of borderline significance that gestational age at presentation and gravidity were associated with outcome at 12 weeks’ gestation. Later gestational age at presentation was associated with a reduced risk of miscarriage with a 1-week increase in gestation associated with the odds of miscarriage reducing by a quarter. Women with gravidity of three or higher had odds of a miscarriage almost three times lower.

**Table III:** Univariable associations between demographic and pre-pregnancy measures and outcome at 12 weeks in the control group.

Variable	Category	Miscarriage n/N (%)	Odds ratio (95% CI)	*P*-value
Age^(*)^	–	–	2.03 (1.25, 3.31)	0.004
Gravidity	1 or 2	23/102 (23%)	1	0.05
	3+	5/54 (9%)	0.35 (0.13, 0.98)	
Parity	0	22/103 (21%)	1	0.13
	1+	6/53 (11%)	0.47 (0.18, 1.24)	
Symptoms	Asymptomatic	8/61 (13%)	1	0.27
	Pain only	6/31 (19%)	1.59 (0.50, 5.07)	
	Bleeding only	5/34 (15%)	1.14 (0.34, 3.81)	
	Pain + bleeding	9/30 (30%)	2.84 (0.97, 8.35)	
Conception method	Spontaneous	12/66 (18%)	1	0.99
	IVF	13/72 (18%)	0.99 (0.42, 2.36)	
	OI	3/18 (17%)	0.90 (0.22, 3.61)	
Gestation^(+)^	–	–	0.74 (0.55, 1.00)	0.05

OI, ovulation induction.

^(*)^ Odds ratio given for a 5-year increase in age.

^(+)^ Gestation at presentation. Odds ratios given for a 1-week increase.

Second stage multivariable analysis of significant factors in the control group is summarized in Table IV. The multivariable results suggested that maternal age, gravidity and gestation at presentation were independently associated with outcome at 12 weeks’ gestation. Older women were at a higher risk of a miscarriage, whilst higher gravidity and later gestation at presentation were associated with a lower risk of miscarriage.

**Table IV:** Multivariable associations between demographic and pre-pregnancy measures and outcome at 12 weeks in the control group.

Variable	Category	Odds ratio (95% CI)	*P*-value
Age^(*)^	–	2.27 (1.34, 3.84)	0.002
Gravidity	1 or 2	1	0.04
	3+	0.31 (0.10, 0.92)	
Gestation^(+)^	–	0.71 (0.51, 0.98)	0.04

^(*)^ Odds ratio given for a 5-year increase in age.

^(+)^ Gestation at presentation. Odds ratios given for a 1-week increase.

Univariable associations in the HP group are outlined in Table V. None of the factors was significantly associated with outcome at 12 weeks’ gestation. The multivariable analysis results are equivalent to those observed in the univariable analysis outlined in Table V. It is noted that the HP group contained a smaller number of women than the control group. Thus, there is less power to detect associations with outcome than observed in the control group.

**Table V:** Univariable associations between demographic and pre-pregnancy measures and outcome at 12 weeks in the study group.

Variable	Category	Miscarriage n/N (%)	Odds ratio (95% CI)	*P*-value
Age^(*)^	–	–	0.46 (0.21, 1.02)	0.06
Gravidity	1 or 2	5/28 (18%)	1	0.91
	3+	4/24 (17%)	0.92 (0.22, 3.90)	
Parity	0	4/28 (14%)	1	0.54
	1+	5/24 (21%)	1.58 (0.37, 6.70)	
Symptoms	Asymptomatic	1/9 (11%)	1	0.88
	Pain only	3/14 (21%)	2.18 (0.19, 25.0)	
	Bleeding only	2/9 (22%)	2.29 (0.17, 31.0)	
	Pain + bleeding	2/14 (14%)	1.33 (0.10, 17.3)	
Conception method	Spontaneous	7/22 (32%)	(#)	0.09
	IVF	2/24 (8%)		
	OI	0/6 (0%)		
Gestation^(+)^	–	–	0.86 (0.55, 1.33)	0.50

OI, ovulation induction.

^(*)^ Odds ratio given for a 5-year increase in age.

^(+)^ Gestation at presentation. Odds ratios given for a 1-week increase.

(#) Unable to calculate odds ratios due to no miscarriages in one group. Analysis using Fisher’s exact test.

## Discussion

Our study showed that in women diagnosed with HP, surgical treatment of extrauterine ectopic pregnancy was not associated with an increased risk of miscarriage of concomitant live normally sited pregnancy compared to controls matched for maternal age, mode of conception and gestational age at presentation. Even when adjusted for factors such as gravidity, parity and symptoms at presentation, there were no statistically significant differences in outcomes between the two groups.

Our study is one of the largest to report on the outcome of live normally sited pregnancies in a population of women with HP and is the first to compare the findings to controls matched for key demographic characteristics. Although the study was retrospective in nature, we had access to computerized patient records including ultrasound images which facilitated data collection, identification of controls and data analysis. However, the number of patients is relatively small, which reflects the rarity of HP. This made it impossible to analyse the outcomes separately in women with uterine ectopic pregnancies. A proportion of HP were excluded from analysis as they did not have a known pregnancy outcome. These women had often continued their care at their local hospitals, and we were unable to complete follow-up. We are therefore unable to comment on whether these women had miscarriages or live births which could affect the results.

Some previous studies on HPs report the rate of loss of concomitant normally sited pregnancies between 26% and 31% (Barrenetxea *et al.*, 2007; Clayton *et al.*, 2007; Talbot *et al.*, 2011; Ko and Cheung, 2012; Li*et al.*, 2016; Xiao *et al.*, 2018), which is much higher than 17% in our study. However, these studies differed from ours as the authors analysed both live normally sited pregnancies and miscarriages at the initial presentation. Similarly, our HP live birth rate of 80% was higher than the 66–69% live birth rate reported by others (Barrenetxea *et al.*, 2007; Clayton*et al.*, 2007; Xiao *et al.*, 2018). Shang *et al.* (2019) reported the pregnancy loss rate as 26%, but after excluding women with miscarriages, the live normally sited pregnancy loss rate was only 12%. Other studies on HPs report a miscarriage rate between 12% and 17% (Yu *et al.*, 2014; Jeon *et al.*, 2016; Na *et al.*, 2018; Wu *et al.*, 2018; Li*et al.*, 2020; Lv *et al.*, 2020) which was similar to our findings.

Surgery is the method of choice for the treatment of HP, as medical therapy with methotrexate is contraindicated in women with live concomitant eutopic pregnancies. In addition, ß-hCG measurements, which are critical in determining the success of conservative management of ectopic pregnancies, cannot be used to monitor the behaviour of HP. In our study, pregnancy outcomes in women treated surgically were not significantly different from controls. This is at odds with the findings of several previous studies which reported miscarriage rates of up to 26% in women who had surgical treatment (Soriano *et al.*, 2010; Eom *et al.*, 2013; Li*et al.*, 2016; Shang *et al.*, 2019). Of note, most of these have included outcomes for all ectopic locations including Caesarean section scar pregnancies, which are treated differently from extrauterine ectopics.

The effect of surgery during pregnancy on the risk of miscarriage is unclear and the maternal condition necessitating surgery could be the main factor associated with risk of miscarriage and preterm labour (Cohen-Kerem *et al.*, 2005; de Bakker *et al.*, 2011; Silvestri *et al.*, 2011; Ball *et al.*, 2019). The advantages of laparoscopic treatment over open procedures are the reduced risk of postoperative adverse events (Liu *et al.*, 2017) and a shorter hospital stay (Cagino *et al.*, 2021). However, a significantly higher risk of adverse foetal outcomes has been reported following laparoscopic versus open surgery for acute appendicitis (McGory *et al.*, 2007; Wilasrusmee*et al.*, 2012; Winter *et al.*, 2017). A more recent meta-analysis, however, showed that the higher risk of adverse outcomes was reported in one study only. When this study was removed from the analysis, there was no significant difference between laparoscopic and open surgery with respect to the risk of foetal loss (Lee *et al.*, 2019).

More than half (58%) of the HP cases in our study were conceived via IVF or ovulation induction, similar to other previous studies (Yu *et al.*, 2014; Jeon*et al.*, 2016; Lyu *et al.*, 2017). A predisposing factor could be tubal damage necessitating ART in the first instance. Tubal dysfunction is thought to be due to changes in tubal transport mechanisms and altered expression of molecules that generally inhibit blastocyst implantation in the Fallopian tube (Refaat *et al.*, 2015). This can be caused by factors such as pelvic infection, history of tubal surgery and ectopic pregnancy (Refaat *et al.*, 2015).

In conclusion, our study has shown that women diagnosed with extrauterine HP and a concomitant live normally sited pregnancy have similar miscarriage and live birth rates following surgical treatment of ectopic pregnancy compared to women in the control group. This information will help clinicians when counselling women diagnosed with HP about the risks associated with surgical treatment. We have also confirmed that ART and history of ectopic pregnancy are significant risk factors for HP and detailed ultrasound examination of the adnexa to rule out an ectopic pregnancy should be carried out in these women whether an eutopic pregnancy is present or not.

## Data availability

The data underlying this article will be shared on reasonable request to the corresponding author.
